# Supplementary material for: Doped Graphene Quantum Dots as Biocompatible Radical Scavenging Agents
Source: Antioxidants (Basel). 2023 Jul 31;12(8):1536. doi: 10.3390/antiox12081536 (PMC10451549; doi:10.3390/antiox12081536)
Supplement: Supplementary file 1 [file antioxidants-12-01536-s001.zip › antioxidants-2506151-supplementary.pdf]

Supporting Information

Doped Graphene Quantum Dots as Biocompatible Radical Scavenging Agents

Adam Bhaloo [1], Steven Nguyen [1], Bong Han Lee [1], Alina Valimukhametova [1], Roberto Gonzalez-Rodriguez [2], Olivia Sottile [1], Abby Dorsky [1] and Anton V. Naumov [1,*]

[1] Department of Physics and Astronomy, Texas Christian University, Fort Worth, TX 76129, USA
[2] Department of Chemistry and Biochemistry, Texas Christian University, Fort Worth, TX 76129, USA
* Correspondence: a.naumov@tcu.edu

a)

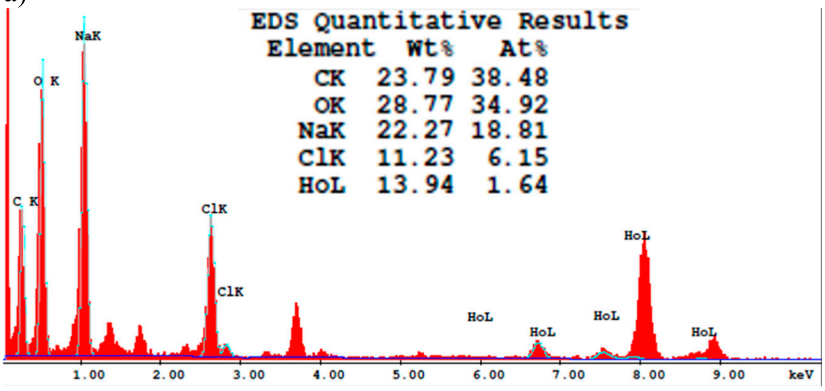

b)

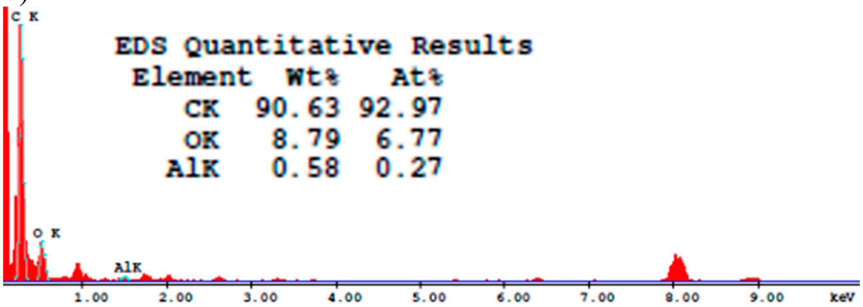

c)

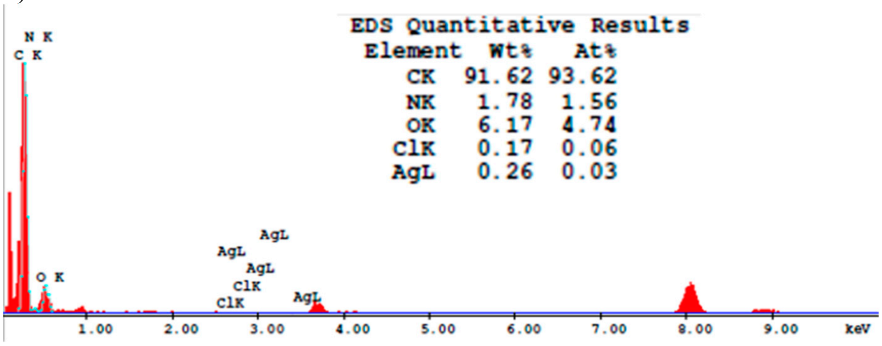

Figure S1. Energy Dispersive X-ray (EDX) spectra, along with weight and atomic percentage information for the listed doped Graphene Quantum Dots (GQDs): (a) Holmium-doped GQDs (Ho-GQDs), (b) Aluminum-doped GQDs (Al-GQDs), (c) Silver-doped GQDs (Ag-GQDs).

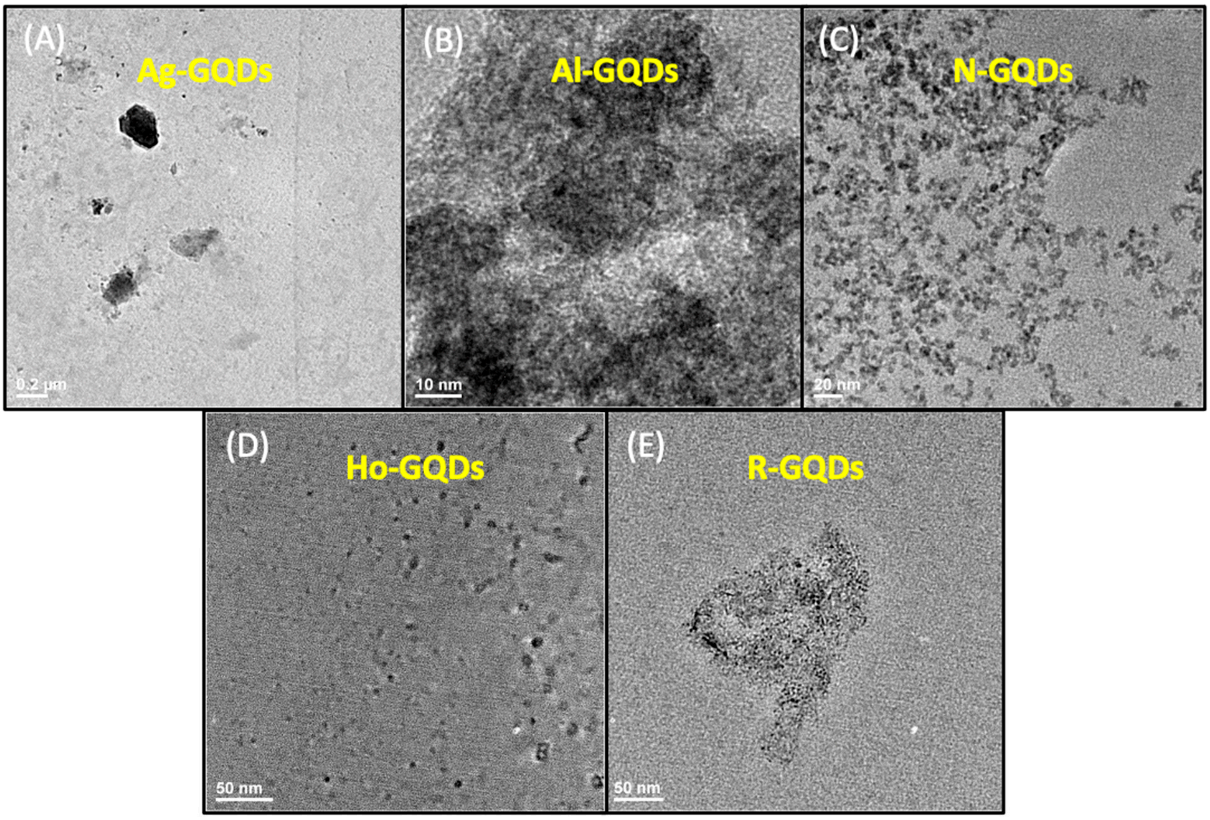

Figure S2. TEM images of (A) Ag-GQDs, (B) Al-GQDs, (C) N-GQDs, (D) Ho-GQDs, and (E) R-GQDs.

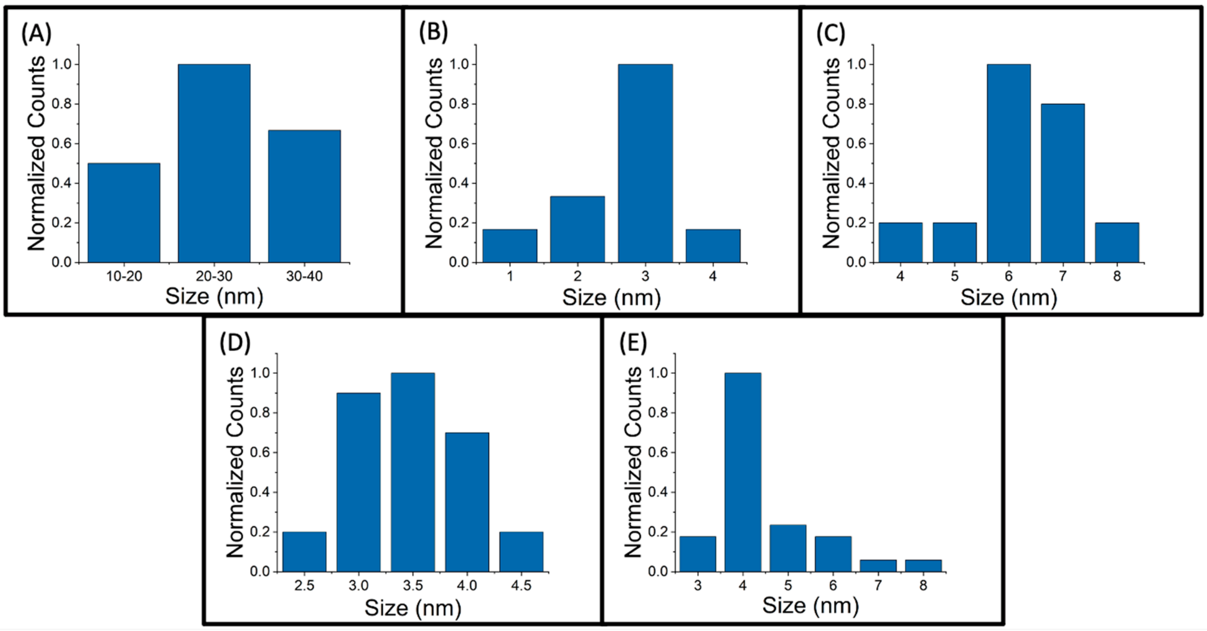

Figure S3. Size distributions of (A) Ag-GQDs (mean at 27.24±5.33 nm), (B) Al-GQDs (mean at 2.79±0.53 nm), (C) N-GQDs (mean at 3.28±0.50 nm), (D) Ho-GQDs (mean at 6.36±1.75 nm), and (E) R-GQDs (mean at 4.48±1.12 nm). The size of each GQD type was calculated by finding the average of all the particle sizes in each TEM image.

a)

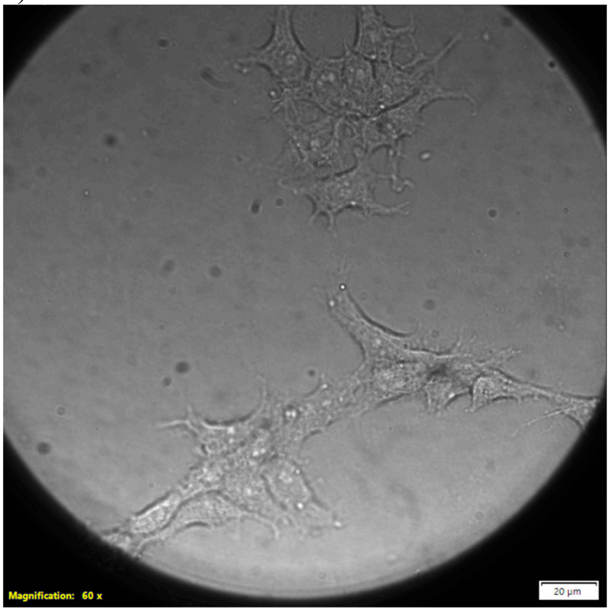

b)

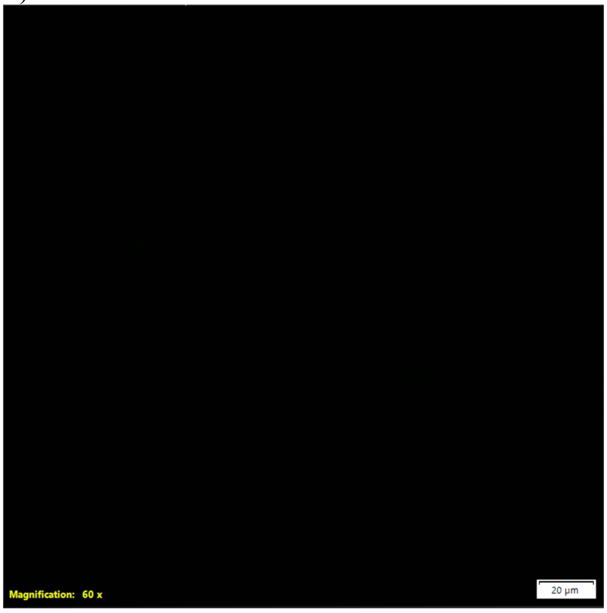

Figure S4. (a) Brightfield and (b) fluorescence images of control cells without GQDs.

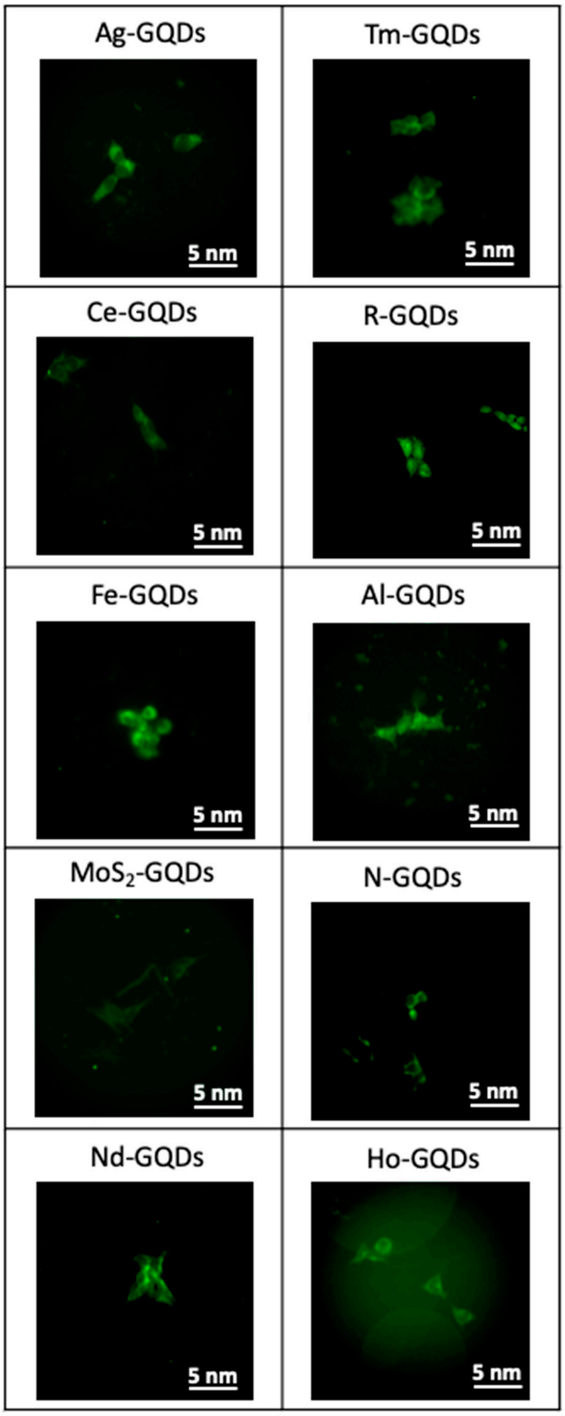

Figure S5. Visible fluorescence confocal images of Ag-, Al-, Ce-, Fe-, MoS_2-, Ho-, N-, Nd-, Tm-, and R- GQDs internalized in HEK-293 cells for 12 hours.

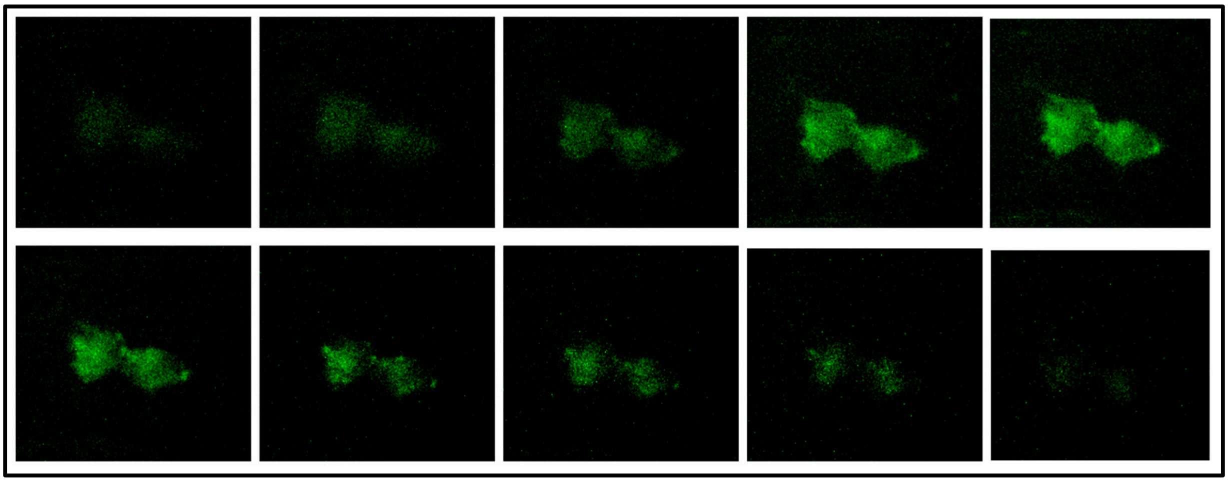

Figure S6. Visible fluorescence confocal z-stack images of N-GQDs internalized within HEK-239 cells for 12 hours. Scale bar: 5 nm.
